# Web GIS in practice II: interactive SVG maps of diagnoses of sexually transmitted diseases by Primary Care Trust in London, 1997 – 2003

**DOI:** 10.1186/1476-072X-4-4

**Published:** 2005-01-18

**Authors:** Maged N Kamel Boulos, Chris Russell, Michael Smith

**Affiliations:** 1 School for Health, University of Bath, Claverton Down, Bath BA2 7AY, UK; 2 Graphical Data Capture Ltd, 3rd Floor, 69 Wilson Street, London EC2A 2BB, UK

## Abstract

**Background:**

The rates of Sexually transmitted diseases (STDs) in England have been rising steadily since the mid 1990s, making them a major public health concern. In 2003, 672,718 people were diagnosed with an STD in England, and around one third of those cases were diagnosed in London.

**Results:**

Using GeoReveal v1.1 for Windows, we produced Web-based interactive choropleth maps of diagnoses of STDs by Primary Care Trust (PCT) in London for the years from 1997 to 2003 . These maps are in Scalable Vector Graphics (SVG) format and require a freely available Adobe SVG browser plug-in to be displayed. They are based on data obtained from the House of Commons Hansard Written Answers for 15 October 2004. They show steadily rising rates of STDs in London over the covered seven-year period. Also, one can clearly see on the maps that PCTs located in central London had the highest numbers of STD diagnoses throughout the mapped seven years. A companion bar chart allows users to instantly compare the STD figure of a given PCT for a given year against the average figure for all 25 mapped PCTs for the same year, and also compare those figures across all seven years. The maps offer users a rich set of useful features and functions, including the ability to change the classification method in use, the number of ranges in the map, and the colour theme, among others.

**Conclusions:**

Wizard-driven tools like GeoReveal have made it very easy to transform complex raw data into valuable decision support information products (interactive Web maps) in very little time and without requiring much expertise. The resultant interactive maps have the potential of further supporting health planners and decision makers in their planning and management tasks by allowing them to graphically interrogate data, instantly spot trends, and make quick and effective visual comparisons of geographically differentiated phenomena between different geographical areas and over time.

SVG makes an ideal format for such maps. SVG is a World Wide Web Consortium non-proprietary, XML-based vector graphics format, and is an extremely powerful alternative to Macromedia^® ^Flash and bitmap graphics.

## Background

Sexually transmitted diseases (STDs) have become a major public health concern in the UK during recent years. The rates of STDs in England have been rising steadily since the mid 1990s. In 2003, the number of STDs in England rose by 4% compared to 2002. Overall, 672,718 people were diagnosed with an STD in England in 2003, and around one third of those cases were diagnosed in the London area alone [1,2].

The House of Commons Hansard Written Answers for 15 October 2004 included an answer by Melanie Johnson MP, Minister for Public Health at the Department of Health, to a question by Sarah Teather MP on "how many cases of diagnosed STDs there were in each Primary Care Trust (PCT) in London in each year since 1997". The answer was provided in the form of a long table showing the figures for 25 PCTs in London (Table 1 – [2]).

**Table 1:** Diagnoses of STDs by PCT in London, 1997 – 2003

**PCT Name**	**1997**	**1998**	**1999**	**2000**	**2001**	**2002**	**2003**
Barking and Dagenham	2,454	2,797	3,020	2,764	2,457	3,461	3,292
Barnet	1,542	1,591	1,546	1,993	2,001	1,899	923
Brent	8,753	8,851	8,724	9,258	9,479	10,156	10,113
Bromley	1,386	1,899	2,287	2,468	3,296	3,683	3,575
Camden	15,714	17,117	17,478	18,750	20,962	22,257	26,987
City and Hackney	14,998	16,595	16,601	16,169	19,171	22,673	21,072
Croydon	4,089	5,546	5,707	6,968	7,805	7,995	7,587
Ealing	1,514	585	1,518	1,687	2,062	2,751	1,832
Enfield	581	1,513	1,168	1,481	1,957	2,129	1,659
Greenwich	4,361	4,314	4,901	6,012	5,170	5,385	6,607
Hammersmith and Fulham	7,838	7,889	8,281	9,556	9,992	4,996	5,723
Haringey	5,029	5,229	5,568	6,344	7,183	6,617	6,379
Hillingdon	1,931	2,387	3,038	3,096	4,131	4,000	3,110
Hounslow	3,270	3,185	3,029	4,101	4,637	5,861	6,473
Islington	5,339	5,552	6,259	5,781	5,309	4,988	6,186
Kensington and Chelsea	11,995	11,636	11,040	13,149	13,301	11,739	12,243
Kingston	1,926	2,443	2,646	3,129	3,474	3,872	4,481
Lambeth	17,865	19,998	21,327	19,971	19,754	22,003	22,209
Lewisham	412	353	346	482	21	-	-
Newham	8,948	9,033	11,214	11,023	12,769	13,200	15,236
Southwark	16,481	17,073	15,232	13,836	16,699	19,618	19,249
Sutton and Merton	10,870	9,110	11,569	13,464	14,229	16,589	17,784
Walthamstow, Leyton and Leytonstone	1,157	1,355	1,726	1,713	2,311	2,433	2,988
Wandsworth	2,629	2,712	3,072	3,004	3,519	4,151	4,199
Westminster	18,639	21,490	21,359	20,503	20,870	18,462	19,657

Diagnoses of sexually transmitted diseases by Primary Care Trust in London, 1997 – 2003 (Source: [2]). Overall figures may be lower than stated for the London region in the annual report because the data presented here have not been imputed. One clinic in each of these PCTs (Barnet; City and Hackney) did not submit all the KC60 returns for 2003. The Alexis Clinic of Lewisham PCT closed in June 2001; no GUM clinics currently open in this PCT.

Though the table presents all the requested data, it remains very difficult for the reader to fully appreciate the patterns and trends buried in them, or make quick and effective comparisons between the figures for different PCTs or between the seven data sets for the years from 1997 to 2003.

Such data patterns, trends and comparisons derived from this Hansard table and other sources, e.g., demographic, deprivation/social exclusion, transport and existing GUM (Genito-Urinary Medicine) clinic data sets, are crucial for the decision maker wanting, for example, to:

- improve access to GUM clinics and make decisions regarding the expansion or closure of existing clinics, or the creation of new ones;

- channel resources and target STD prevention programmes to areas with the most need, or scale such programmes according to the magnitude of the problem in different areas (this is especially important in a climate of finite resources); and/or

- monitor the impact of such programmes in a given area over time.

In this paper, we describe a much better way of presenting the same Hansard table data in the form of interactive Web maps in Scalable Vector Graphics (SVG) format to further support health planners and decision makers in their planning and management tasks.

## Results

**Figure 1:**
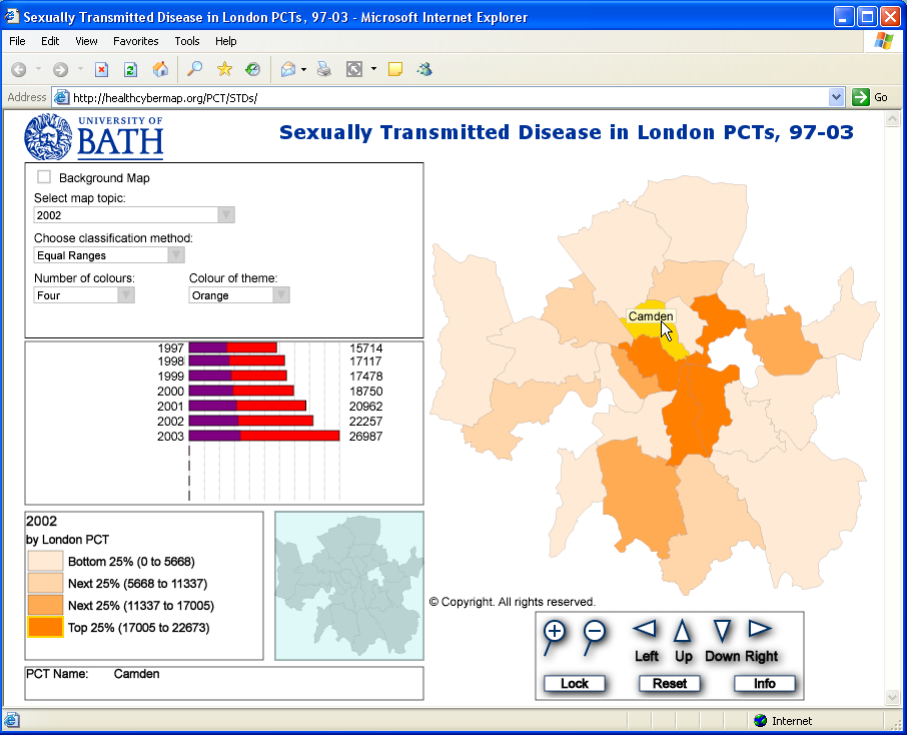
**Screenshot from our interactive Web maps of STD diagnoses by London PCT, 1997 – 2003. **Screenshot from our Web-based interactive choropleth maps of diagnoses of STDs by PCT in London for the years from 1997 to 2003 . The map shown in this screenshot is for the year 2002, with Camden PCT highlighted in yellow. The bar chart ('Chart Panel') on the left shows steadily rising STD rates in Camden PCT over the covered seven-year period. Camden's rates are well above the average for all 25 mapped PCTs over the same period (the purple portions of the bars represent the average for all PCTs). The maps require the free Adobe SVG Viewer . Visitors will be automatically prompted to download it on their first visit to the site, if they don't already have it installed on their machine. Scripting must also be enabled in Internet Explorer.

Using GeoReveal, a tool from Graphical Data Capture Ltd ( – see 'Methods' section below), we produced Web-based interactive choropleth maps of diagnoses of STDs by PCT in London for the years from 1997 to 2003, which readers can browse at  (Figure 1). These maps and companion bar chart ('Chart Panel') are based on the data in Table 1, and show steadily rising rates of STDs in London over the covered seven-year period. Also, one can clearly see on the maps that PCTs located in central London, e.g., Camden PCT and Lambeth PCT, had the highest numbers of STD diagnoses throughout the seven years from 1997 to 2003.

As the mouse cursor is moved around the main map window, the 'Chart Panel' changes to display statistical data about the currently highlighted PCT. Each row of the bar chart represents data for one year and has two bars; a red bar that shows the value for the highlighted PCT, and a transparent blue bar which shows a mean value of this piece of data (or year) for all 25 mapped PCTs (when both bars overlap, a purple colour is produced – Figure 1).

**Figure 2:**
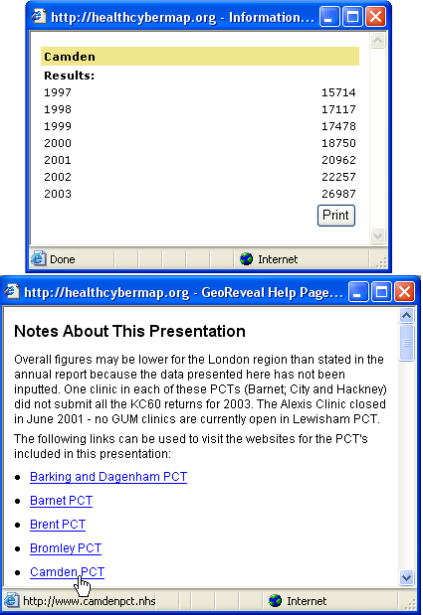
**Screenshots of two information boxes from our interactive Web map interface. **Upper information box: clicking a PCT area on the map ('Camden PCT' for this screenshot) will display this pop-up box with all available data for that PCT. Lower information box: clicking the 'Info' button (shown in Figure 1 – bottom right 'Navigation Panel') will open this pop-up box with extra information about the maps, links to the Web sites of London PCTs, and detailed help about the map interface.

Additionally, clicking a PCT area on the map will display an information box with all available data for that PCT (Figure 2).

An 'Options Panel' controls the data that are shown in the map window. Users can tick the 'Background Map' box to add a raster background map of London to the main map. They can use the 'Choose Classification Method' list to select how the data are mapped; with equal ranges, equal counts or by highlighting the highest values. A 'Number of Colours' list allows users to select how many classes or ranges are used in the choropleth map (two to five ranges). Using the 'Select Map Topic' list, users can select the topic that is shown on the main map (a year from 1997 to 2003). Finally, users can select from the 'Colour of Theme' list the colour theme that is used in the main map (orange, green, or blue).

Map zooming, panning, MapTips (displaying PCT names), and a dynamic legend are available. An overview map shows a miniature version of the full extent map. When the user zooms in, a rectangle on the overview map highlights the area that is currently being displayed in the main map window. The user can click and drag this rectangle to change the view in the main map window. After zooming into the main map, users can use the 'Reset' button to return to the full extent of the main map.

An 'Info' button is also available. Clicking this button will open a pop-up window with extra information about the maps, links to the Web sites of London PCTs, and detailed help about the map interface (Figure 2).

## Discussion

### From complex raw data to valuable decision support information

Turning raw tabular data into much more useful and accessible visual information in the form of interactive Web maps is much needed to support and empower decision makers, and even members of the general public. Such maps help us understand the relationships, patterns and trends buried in the original data sets and also enable instant visual comparisons to be made between different geographical areas and over time (when data sets for successive periods of time are available) [3].

We believe this transformation of raw data into valuable decision support information is very evident in the London STD example described in this paper. Readers only have to compare the original Hansard table (Table 1) with the corresponding interactive Web maps we have produced  to see the difference for themselves and appreciate the value of interactive maps.

### SVG: an ideal format for interactive Web maps

SVG is a non-proprietary language for describing rich, stylable two-dimensional graphics and graphical applications in XML (eXtensible Markup Language). SVG is fully endorsed by the W3C (World Wide Web Consortium – ). It is rapidly becoming a popular choice for delivering interactive Web maps, being designed to work effectively across platforms, output resolutions, colour spaces, and a range of available bandwidths. It offers a rich modern graphics format providing the ability for better map display, and advanced graphical features such as transparency, arbitrary geometry, filter effects (shadows, lighting effects, etc.), scripting, and animation [4].

All these features have made SVG a direct competitor to the proprietary Macromedia^® ^Flash format [5]. Vector-based images (describing shapes and paths), such as those in SVG and SWF (Macromedia^® ^Shockwave/Flash File) formats, will keep their sharp character when enlarged, while raster-based images (storing information about each and every pixel in the image), such as those saved in GIF (Graphics Interchange Format) or JPEG (Joint Photographic Experts Group) formats, will show jagged edges.

A free SVG Web browser plug-in is available from Adobe for different platforms (Adobe SVG Viewer – ), in the same way the free Adobe Reader software is available for rendering PDF (Portable Document Format) files.

Besides the example described in this paper, other examples of SVG interactive Web maps in the health arena include the Office for National Statistics' England and Wales 2001 Census Key Statistics maps  and 2001 Area Classification for Health Areas maps , and Leeds Interactive Health Atlas .

### Tools for producing interactive SVG and Flash maps from desktop GIS projects

Besides GeoReveal , the tool we have used to produce the maps described in this paper (see 'Methods' section below), other SVG/Flash mapping tools available today for publishing maps created in desktop GIS (Geographic Information Systems) include GéoClip , SVGMapMaker , MapViewSVG , and SVGMapper . The latter two tools (MapViewSVG and SVGMapper) are specific to ESRI ArcView GIS. Table 2 provides an overview of the features of GeoReveal, GéoClip, and SVGMapMaker.

**Table 2:** Overview of the features of GeoReveal, GéoClip, and SVGMapMaker

**GeoReveal**	**GéoClip**	**SVGMapMaker**
GeoReveal is a powerful .NET application that combines geography and statistics to produce revealing interactive graphics for the Web. It produces fully interactive geo-statistical presentations. There is no requirement for a GIS. GeoReveal uses MID/MIF files that can be exported from practically any GIS. Other information: - Unlimited data fields and includes a comprehensive set of pre-defined templates - Features a simple wizard driven interface and an advanced set of menus for users who need to fine tune the finished output - Generates SVG data, overlay boundaries, overview and background maps. For UK Local Government users, SVG is also an e-GIF (e-Goverment Interoperability Framework) compliant format - Presentations can include interactive charts that change to display statistics about the currently selected map region or area - Map display can be changed after generation, i.e., ability to change map topic, map colour, number of ranges, and classification method - Includes pre-prepared UK census boundary data and sample projects	GéoClip is a MapBasic program that takes TAB file information and turns it into interactive Macromedia^® ^Flash presentations. There is also a version for ESRI ArcGIS. GéoClip does not currently have an SVG component. Other information: - Written in MapBasic and therefore dependent on having a MapInfo Pro licence - Does not offer a very intuitive interface - Limited to ten data fields and to only one page layout - Users cannot remove the GéoClip logo/branding from the map page - Can only produce Flash (proprietary format, not e-GIF compliant) and not SVG (open format) - Some of its unique features like adjustable ranges and multiple themes are planned for the next release of GeoReveal	SVGMapMaker is a MapBasic program that takes TAB file information and uses it to replicate limited GIS functionality in a browser window. It cannot be considered as a geo-statistical tool, as the level of interactivity is limited. For example, there are no interactive charts as provided in GeoReveal. Other information: - Written in MapBasic and therefore dependent on having a MapInfo Pro licence - Limited interface – no ability to change map topic and no statistical delivery - Map display cannot be changed after generation, i.e., no ability to change map colour, number of ranges, and classification method

A quick comparison of three tools available today for producing interactive SVG and Flash maps from desktop GIS projects: GeoReveal (from Graphical Data Capture Ltd, London, UK – ), GéoClip (from eMc3, a young company based in Toulouse, France – ), and SVGMapMaker (from TETRAD Computer Applications Inc, Bellingham, WA, USA – ).

## Conclusions

Using GeoReveal v1.1 for Windows, we produced Web-based interactive choropleth maps of diagnoses of STDs by PCT in London for the years from 1997 to 2003 . These maps are in SVG format and require a freely available Adobe SVG browser plug-in to be displayed. They are based on data obtained from the House of Commons Hansard Written Answers for 15 October 2004. They show steadily rising rates of STDs in London over the covered seven-year period. Also, one can clearly see on the maps that PCTs located in central London had the highest numbers of STD diagnoses throughout the mapped seven years. A companion bar chart allows users to instantly compare the STD figure of a given PCT for a given year against the average figure for all 25 mapped PCTs for the same year, and also compare those figures across all seven years. The maps offer users a rich set of useful features and functions, including the ability to change the classification method in use, the number of ranges in the map, and the colour theme, among others.

We also presented a quick review of some of the tools available today for creating interactive vector graphics maps from desktop GIS projects.

Wizard-driven tools like GeoReveal have made it very easy to transform complex raw data into valuable decision support information products (interactive Web maps) in very little time and without requiring much expertise. The resultant interactive maps have the potential of further supporting health planners and decision makers in their planning and management tasks by allowing them to graphically interrogate data, instantly spot trends, and make quick and effective visual comparisons of geographically differentiated phenomena between different geographical areas and over time.

SVG makes an ideal format for such maps. SVG is a W3C non-proprietary, XML-based vector graphics format, and is an extremely powerful alternative to Macromedia^® ^Flash and bitmap graphics.

## Methods

We used GeoReveal v1.1 for Windows to create the interactive SVG maps described in this paper. GeoReveal runs under Windows 98/NT/2000/XP and requires Microsoft^® ^.NET framework v1.1 to be installed on the production machine.

We started by extracting London PCT boundaries from a larger data set of all England (2001 Census PCT – post April 2002 change), which is the copyright of the Crown/Ordnance Survey , and is freely available in both ArcView and MapInfo formats to the UK academic community from EDINA UKBORDERS service with the support of the ESRC and JISC . We also prepared a spreadsheet containing data about the number of STDs recorded in each London PCT between 1997 and 2003 using data from [2]. The two files were merged using MapInfo Professional v7.5 , creating a MapInfo .TAB file. This file was exported to MID/MIF format, the format that files need to be in, in order to be used by GeoReveal.

We created our presentation (the interactive SVG Web maps) using the GeoReveal Wizard; an eight-step process that allows users to create a fully interactive SVG page.

### Introduction: setting the output directory

**Figure 3:**
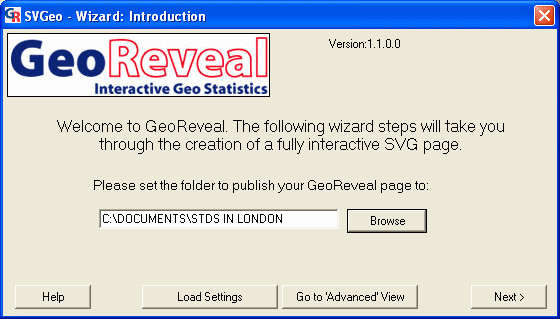
**Screenshot of the 'Introduction' dialog. **Screenshot of the 'Introduction' dialog in GeoReveal Wizard. For a detailed description of the functions available in this dialog, please refer to the 'Methods' section > 'Introduction: setting the output directory'.

The first Wizard dialog that must be completed is the 'Introduction' dialog, which is used to specify the directory to which the final GeoReveal output files will be saved (Figure 3).

To select the output directory, we clicked the 'Browse' button and in the resulting file browse dialog, we selected the required directory and clicked 'OK'. To move to the next step, we clicked 'Next'.

### Wizard step 1: choosing the template

**Figure 4:**
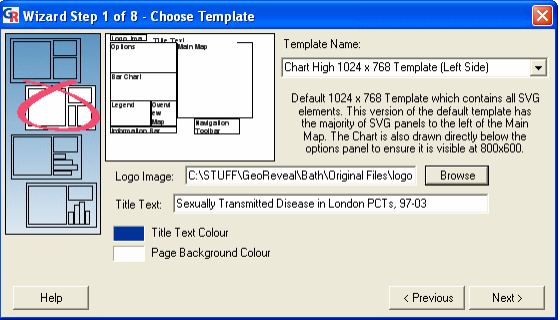
**Screenshot of the 'Choose Template' dialog. **Screenshot of the 'Choose Template' dialog in GeoReveal Wizard (Wizard Step 1 of 8). For a detailed description of the functions available in this dialog, please refer to the 'Methods' section > 'Wizard step 1: choosing the template'.

The first Wizard step is the 'Choose Template' dialog (Figure 4). This dialog is used to specify general settings for a GeoReveal presentation.

We selected the template to be used. Ten templates are provided; four that include a bar chart, four that include a pie chart and two that have no chart. With our London PCT STD data, the most appropriate is a bar chart template.

We selected a logo image to be added to the top-left corner of our GeoReveal page. We clicked the 'Browse' button and selected the required file – in this instance, a University of Bath logo has been used.

We then specified the title of the GeoReveal page. This title is placed next to the logo at the top of the output page (see the logo and title at .

Finally, we selected the title colour and page background colour. To move to the next step, we clicked 'Next'.

### Wizard step 2: selecting the main map data

**Figure 5:**
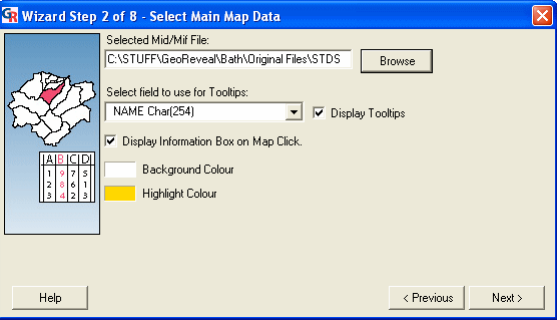
**Screenshot of the 'Select Main Map Data' dialog. **Screenshot of the 'Select Main Map Data' dialog in GeoReveal Wizard (Wizard Step 2 of 8). For a detailed description of the functions available in this dialog, please refer to the 'Methods' section > 'Wizard step 2: selecting the main map data'.

The second step is the 'Select Main Map Data' dialog (Figure 5). This dialog is used to specify settings for the main GeoReveal map.

We selected the MID/MIF file that contains the statistics that will form the main map in our GeoReveal page by clicking the 'Browse' button and choosing the required file.

We selected the field from this MID/MIF file that will be used for ToolTips. A ToolTip (or MapTip) is the piece of text that is displayed when the mouse cursor is hovered over a region in the map. In this instance, the PCT Name field has been selected.

Users can also choose to turn the information box on or off. When it is turned on, a user can click a region in the map to display a dialog containing all information held within the MID/MIF file about that region (Figure 2).

Finally, we selected the background colour and highlight colour for the map. The highlight colour is the colour a map region (an individual PCT area in our case) will be displayed in when the mouse cursor hovers over it. To move to the next step, we clicked 'Next'.

### Wizard step 3: selecting the overview map data

**Figure 6:**
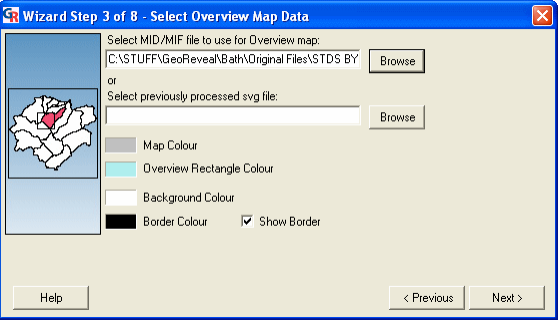
**Screenshot of the 'Select Overview Map Data' dialog. **Screenshot of the 'Select Overview Map Data' dialog in GeoReveal Wizard (Wizard Step 3 of 8). For a detailed description of the functions available in this dialog, please refer to the 'Methods' section > 'Wizard step 3: selecting the overview map data'.

The third step is the 'Select Overview Map Data' dialog (Figure 6). This dialog is used to specify settings for the GeoReveal overview map.

We selected the MID/MIF file to be used for the overview map by clicking the 'Browse' button and selecting the required file. It is possible to use the same MID/MIF file for both the main and overview maps.

We selected the overview map colour and the overview rectangle colour (this rectangle shows a user where they are currently zoomed in on the main map). Additionally, users can select the background colour and border colour for the overview map panel. To move to the next step, we clicked 'Next'.

### Wizard step 4: selecting options controls

**Figure 7:**
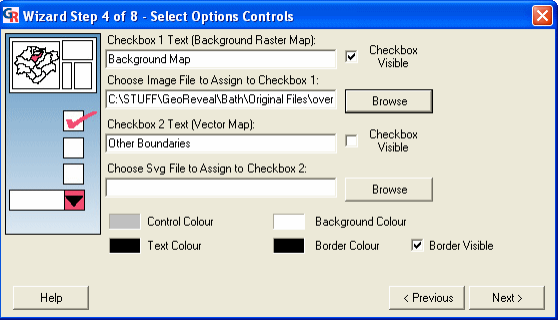
**Screenshot of the 'Select Options Controls' dialog. **Screenshot of the 'Select Options Controls' dialog in GeoReveal Wizard (Wizard Step 4 of 8). For a detailed description of the functions available in this dialog, please refer to the 'Methods' section > 'Wizard step 4: selecting options controls'.

The fourth step is the 'Select Options Controls' dialog (Figure 7). This dialog is used to specify settings for the 'Options Panel' in the presentation.

First, we selected the background map image that is to be used. We ticked the top 'Checkbox Visible' box to activate the background map option and then clicked the top 'Browse' button to select the file that will be used. This file must be a JPEG or GIF image and information is needed about its width and height in real terms, and also the bounding coordinates.

**Figure 8:**
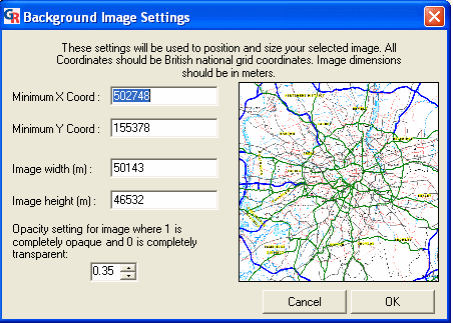
**Screenshot of the 'Background Image Settings' dialog. **Screenshot of the 'Background Image Settings' dialog in GeoReveal Wizard. For a detailed description of the functions available in this dialog, please refer to the 'Methods' section > 'Wizard step 4: selecting options controls'.

Once the required file is selected, the 'Background Image Settings' dialog is displayed (Figure 8). In this dialog, we entered the bounding coordinates of our selected image, the height and width of the image in real terms, and specified how opaque the background map will be in the presentation. We then clicked 'OK' to confirm and return to the previous dialog (Figure 7).

In the 'Select Options Controls' dialog, it is also possible to:

- enable a vector map option; and

- select the text colour, border colour, background colour and control colour (the colour in which the fields are rendered) for the 'Options Panel'.

To move to the next step, we clicked 'Next'.

### Wizard step 5: setting the bar chart

**Figure 9:**
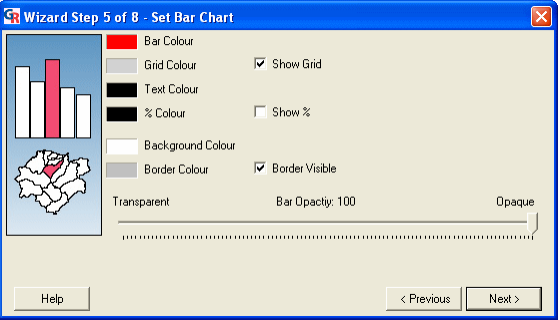
**Screenshot of the 'Set Bar Chart' dialog. **Screenshot of the 'Set Bar Chart' dialog in GeoReveal Wizard (Wizard Step 5 of 8). For a detailed description of the functions available in this dialog, please refer to the 'Methods' section > 'Wizard step 5: setting the bar chart'.

The fifth step is the 'Set Bar Chart' dialog (Figure 9). This dialog is used to specify settings for the bar chart in the presentation. This dialog will differ depending on the template that was selected. In this dialog, one can:

- specify the bar colour, grid colour (if the grid is enabled), text colour, background colour and border colour;

- add or remove a % sign to the figures that are shown at the end of each bar. As the data being used in this presentation are absolute, the 'Show %' box should be unchecked; and

- specify the opacity of the bars in the bar chart.

After specifying our settings for the bar chart, we clicked 'Next' to move to the next step.

### Wizard step 6: setting the legend

**Figure 10:**
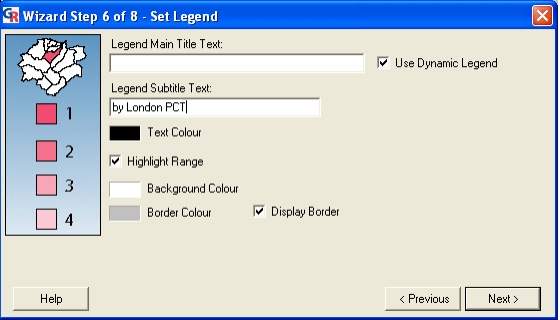
**Screenshot of the 'Set Legend' dialog. **Screenshot of the 'Set Legend' dialog in GeoReveal Wizard (Wizard Step 6 of 8). For a detailed description of the functions available in this dialog, please refer to the 'Methods' section > 'Wizard step 6: setting the legend'.

The sixth step is the 'Set Legend' dialog (Figure 10). This dialog is used to specify settings for the legend panel in the presentation. Users can:

- specify a title and subtitle for the legend. Rather than specifying a main title, we checked the 'Use Dynamic Legend' box. When this box is checked, the title will be determined by the topic that the GeoReveal map is based on (a year from 1997 to 2003 in our case);

- enable or disable the 'Highlight Range' option. If this option is enabled (as is the case in our presentation), when the mouse is hovered over a map region (PCT), the legend range that this region falls into will be highlighted; and

- specify the text colour, background colour and border colour for the legend panel.

After specifying our settings for the legend, we clicked 'Next' to move to the next step.

### Wizard step 7: setting the 'Information Panel'

**Figure 11:**
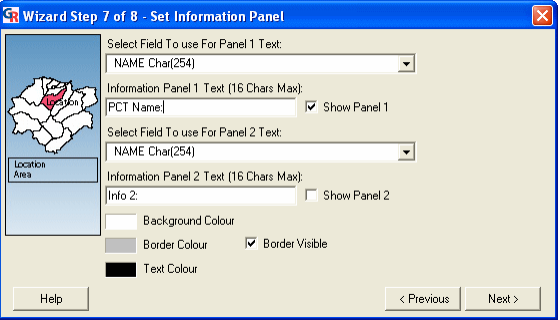
**Screenshot of the 'Set Information Panel' dialog. **Screenshot of the 'Set Information Panel' dialog in GeoReveal Wizard (Wizard Step 7 of 8). For a detailed description of the functions available in this dialog, please refer to the 'Methods' section > 'Wizard step 7: setting the 'Information Panel".

The seventh step is the 'Set Information Panel' dialog (Figure 11). This dialog is used to specify settings for the 'Information Panel' in the presentation. In the presentation, this panel displays information about the region in the map (PCT) that the mouse cursor is currently over. Users can:

- enable or disable each of the information panels and select the fields from the MID/MIF file that will be used to populate each panel. A title can also be entered for each panel (in our instance, Panel 1 was enabled, has been given the title 'PCT Name' and will display the PCT Name field from the MID/MIF file); and

- specify the text colour, background colour and border colour for the 'Information Panel'.

We then clicked 'Next' to move to the next step.

### Wizard step 8: setting the 'Navigation Panel'

**Figure 12:**
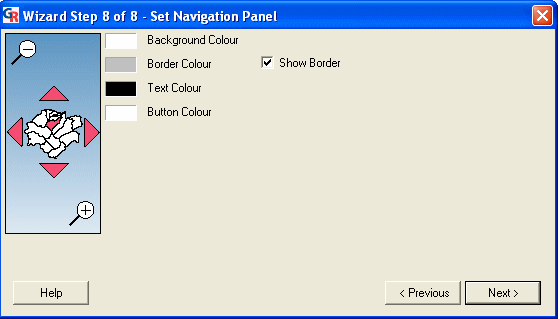
**Screenshot of the 'Set Navigation Panel' dialog. **Screenshot of the 'Set Navigation Panel' dialog in GeoReveal Wizard (Wizard Step 8 of 8). For a detailed description of the functions available in this dialog, please refer to the 'Methods' section > 'Wizard step 8: setting the 'Navigation Panel".

The eighth step is the 'Set Navigation Panel' dialog (Figure 12). This dialog is used to specify settings for the 'Navigation Panel' in the presentation: the text colour, background colour, border colour and button colour.

We then clicked 'Next' to move to the final step.

### Generating the presentation

**Figure 13:**
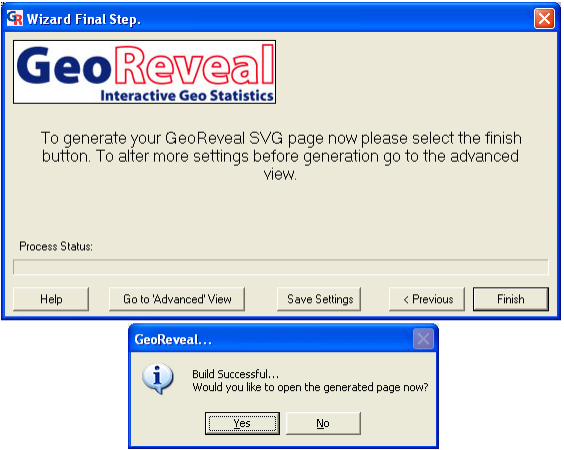
**Screenshots of the 'Wizard Final Step' dialog and final 'Build Successful' message box. **Screenshots of the 'Wizard Final Step' dialog and final 'Build Successful' message box in GeoReveal Wizard. For a detailed description of the functions available in this dialog, please refer to the 'Methods' section > 'Generating the presentation'.

Lastly, we saved the settings for our presentation and generated an initial presentation page (Figure 13). The 'Wizard Final Step' dialog enables users to:

- save the settings they have just made by clicking 'Save Settings', then in the resulting dialog, browsing to the required directory and clicking 'Save'. The saved settings file enables users to restore their settings and edit the presentation at a later date; and

- generate the presentation by clicking 'Finish'. A message is displayed to confirm that the presentation has been successfully generated (Figure 13). Clicking 'Yes' on the message box will open the page in Internet Explorer.

When the presentation is created, the 'Advanced View' window is also opened. This can be used to add the finishing touches to the presentation. It contains all the options that the wizard does, along with a few advanced options.

### 'Advanced View': 'Legend' tab

**Figure 14:**
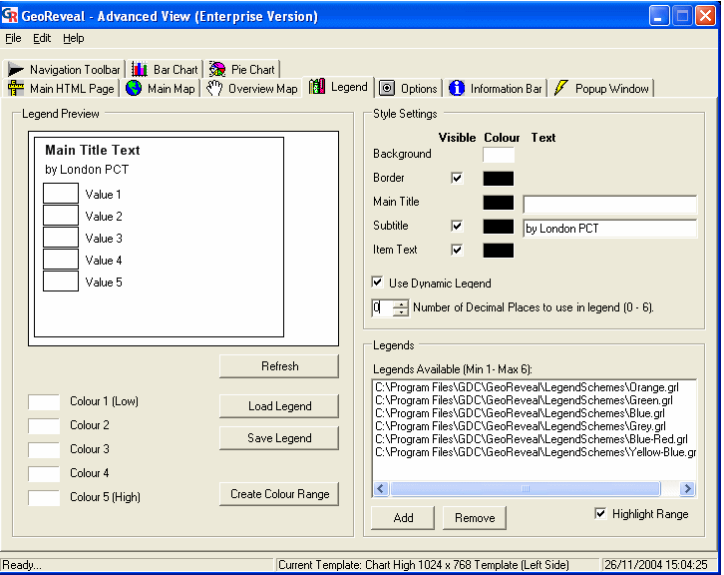
**Screenshot of the 'Legend' tab in the 'Advanced View' window. **Screenshot of the 'Legend' tab in the 'Advanced View' window in GeoReveal Wizard. For a detailed description of the functions available in this tab, please refer to the 'Methods' section > "Advanced View': 'Legend' tab'.

The 'Legend' tab can be used to select the legend colour schemes that will be available (Figure 14). In this tab, users can add and remove colour schemes using the 'Add' and 'Remove' buttons, and ensure that the legend to be first loaded is at the top. Legend schemes are provided with the GeoReveal installation, and additional ones can be created using this tab. Moreover, the number of decimal places used in the legend can be changed. For this presentation, it has been set to 0.

### 'Advanced View': 'Bar Chart' tab

**Figure 15:**
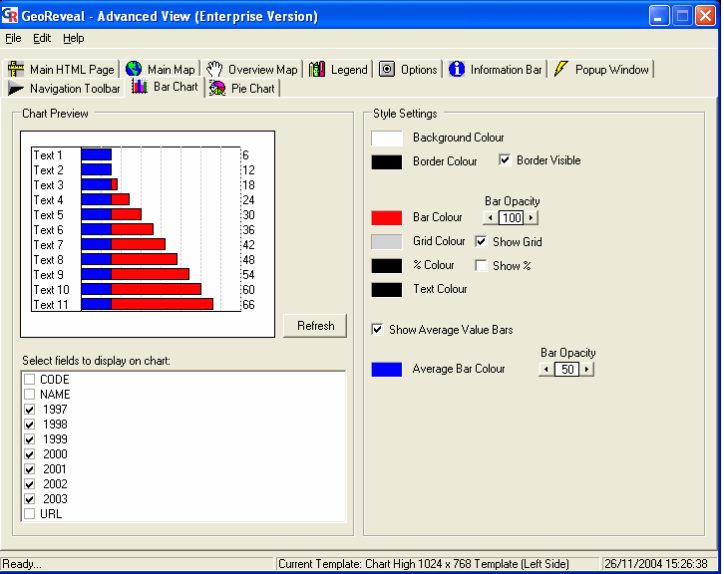
**Screenshot of the 'Bar Chart' tab in the 'Advanced View' window. **Screenshot of the 'Bar Chart' tab in the 'Advanced View' window in GeoReveal Wizard. For a detailed description of the functions available in this tab, please refer to the 'Methods' section > "Advanced View': 'Bar Chart' tab'.

The 'Bar Chart' tab can be used to edit the bar chart (Figure 15). It is possible to add average value bars to the bar chart. When enabled, a second bar is added to each row of the chart which shows the mean value of all the data in that field, as opposed to the first set of bars showing values for the currently selected map region (PCT) alone. To enable this:

- we ticked the 'Show Average Value Bars' box to add average value bars to the bar chart; and

- set the average bar colour and specified the opacity of the bars.

### 'Advanced View': 'Navigation Toolbar' tab

**Figure 16:**
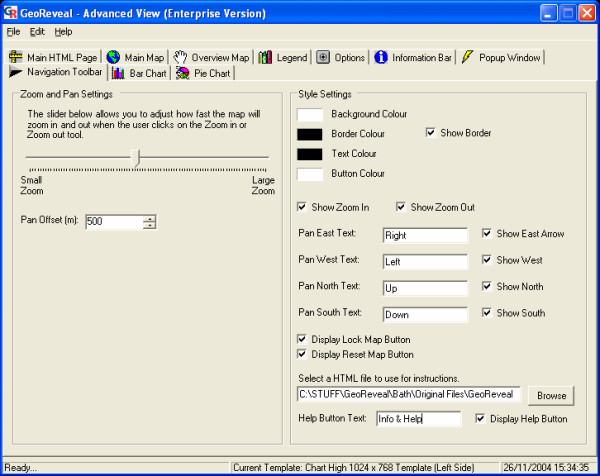
**Screenshot of the 'Navigation Toolbar' tab in the 'Advanced View' window. **Screenshot of the 'Navigation Toolbar' tab in the 'Advanced View' window in GeoReveal Wizard. For a detailed description of the functions available in this tab, please refer to the 'Methods' section > "Advanced View': 'Navigation Toolbar' tab'.

Finally, we used the 'Navigation Toolbar' tab to edit the 'Navigation Panel' (Figure 16). In this tab, it is possible to add a button to the 'Navigation Panel', which when clicked, will open a simple HTML (HyperText Markup Language) page that contains information on how the GeoReveal presentation can be used and additional information about what it shows. To do this:

- we enabled the help button by ticking the 'Display Help Button' box;

- entered the text that will be shown on the button. In this instance, it will show 'Info'; and

- selected the help file that will be used by clicking the 'Browse' button and selecting the required file. In this instance, an HTML document was created that contains hyperlinks to the PCT Web sites, and information about using the presentation (see lower information box in Figure 2).

### Regenerating the presentation

We then resaved our settings by going to 'File' > 'Save Settings'. In the resulting dialog, we browsed to the required directory and clicked 'Save'. The presentation was regenerated by clicking 'File' > 'Generate SVG Page'. Finally, we uploaded all the generated final page files to our Web server ( – Figure 1).

## Competing interests

CR and MS work for GDC (Graphical Data Capture Ltd), the company that produces GeoReveal.

## Authors' contributions

All three authors contributed equally to this paper.
